# Probiotic Yeasts and Bacteria: A Complementary Approach to Gut Health

**DOI:** 10.3390/microorganisms14081763

**Published:** 2026-08-11

**Authors:** Arrigo F. G. Cicero, Cecilia Bartoli, Carla Lluís-Ganella, Paolo Fabrizzi

**Affiliations:** 1 Medical and Surgical Sciences Department, Alma Mater Studiorum University of Bologna, 40130 Bologna, Italy; arrigo.cicero@unibo.it; 2 Medical Affairs, Menarini, Via Sette Santi 1-3, 50131 Florence, Italy; pfabrizzi@menarini.com; 3 MedAffairs Experts, Pl. de Catalunya, 1, 08002 Barcelona, Spain; carla.lluis@medaffairsexperts.com

**Keywords:** Gut microbiota dysbiosis, probiotics, *Saccharomyces boulardii*, *Kluyveromyces marxianus*, synbiotics, intestinal barrier, microbiota homeostasis

## Abstract

Probiotics are live microorganisms that, when administered in adequate amounts, confer a health benefit to the host. Research has traditionally focused on bacterial species, particularly *Lacticaseibacillus* and *Bifidobacterium*, whose mechanisms are well-characterised. However, probiotic yeasts represent a functionally distinct category, with *Saccharomyces boulardii* as the most studied representative and *Kluyveromyces marxianus* as an emerging candidate. Bacterial and yeast probiotics differ in cell biology, antibiotic susceptibility, immune receptor engagement, and metabolic output. Bacterial strains provide mucosal adhesion and direct short-chain fatty acid (SCFA) generation but are susceptible to antibacterial therapy. Yeast strains are intrinsically antibiotic-resistant, offer enzymatic activities such as β-galactosidase and glycosidases, and exert indirect bifidogenic effects that amplify luminal SCFA concentrations beyond what either category achieves alone. These non-overlapping profiles support a complementary rather than competitive conceptualisation of their use. When combined with prebiotic substrates such as fructooligosaccharides or galactooligosaccharides, this integrated approach addresses the principal axes of intestinal homeostasis more comprehensively than any single-organism intervention. Prospective randomised trials are needed to determine whether this mechanistic complementarity translates into additive or synergistic clinical benefit. This review synthesises the available evidence and proposes a framework for their complementary use. The proposed framework is hypothesis-generating: it rests largely on mechanistic and preclinical data, and its clinical validity requires confirmation in adequately powered controlled trials.

## 1. Introduction

The human intestine contains around 10^14^ microorganisms representing thousands of bacterial, archaeal, fungal, and viral species that together form the gut microbiota, a complex ecosystem with pivotal metabolic, trophic, and protective functions that extend beyond simple digestion [1]. Through the gut–brain axis, this microbiota communicates bidirectionally with the central nervous system via neuroendocrine and immune pathways, including the production of neurotransmitter precursors, modulation of hormonal signals, and regulation of inflammatory responses [2]. In a state of eubiosis, a diverse community dominated by Firmicutes and Bacteroidetes, which together account for about 90% of the bacterial population [3], functions as an integral physiological component of the host, supporting metabolic homeostasis, epithelial renewal, mucosal barrier integrity, and immune calibration. Microbial composition is substantially established during the first two to three years of life and is subsequently shaped by diet, antibiotic exposure, lifestyle, and host genetic factors [4]. The stability of this ecosystem across the human lifespan is not merely a passive consequence of microbial colonisation but the product of dynamic interactions between commensal organisms, host epithelial cells, and immune effectors [5]. Beyond this bacterial majority, the gut also harbours a fungal community, the mycobiota, whose ecological interactions with resident bacteria are only beginning to be characterised; these trans-kingdom relationships represent an under-examined dimension of intestinal homeostasis and provide part of the conceptual rationale for the present synthesis [6,7,8]. Under physiological conditions, fungi, bacteria, and viruses in the gut maintain a stable yet dynamic relationship with one another that may be neutral, synergistic, or antagonistic [9]. For example, the common fungus *Candida albicans* interacts with several bacterial taxa, including *Clostridioides difficile* and *Enterococcus faecalis*, altering their assembly and function through cell-membrane contact, competition or cooperation for nutrients, and the production of secondary metabolites and antimicrobial peptides [9].

The intestinal microbiota performs several indispensable metabolic functions for the host. Among the most relevant is the anaerobic fermentation of non-digestible dietary fibres to generate short-chain fatty acids (SCFAs), such as acetate, propionate, and butyrate. Commensal bacteria also contribute to the synthesis of vitamins K [10] and B12 [11] and facilitate the intestinal absorption of minerals including calcium, zinc, iron, and manganese [12,13]. In this metabolic network, butyrate is the main energy substrate for colonocytes and is key for preserving epithelial integrity, whereas acetate and propionate exert predominantly immunoregulatory effects on both systemic and mucosal immunity. SCFAs additionally modulate several aspects of intestinal homeostasis by lowering luminal pH, enhancing mucus production and thickness, and stabilising tight junction proteins through activation of G-protein-coupled receptors such as GPR43, GPR41, and GPR109a on epithelial and immune cells, thereby promoting an environment that supports both effective barrier function and immune tolerance [14].

Beyond metabolic support, the microbiota participates in continuous immunological education: commensal microbial structures engage pattern-recognition receptors on epithelial and dendritic cells, driving the differentiation of regulatory T cells that secrete interleukin-10 and transforming growth factor-β, sustaining the mucosal tolerance that prevents inappropriate inflammatory responses to harmless luminal antigens [15,16,17]. When this balanced ecosystem is disrupted by antibiotic exposure, dietary insufficiency, or other exogenous stressors, the resulting dysbiosis impairs SCFAs production, weakens epithelial tight junction integrity, and perturbs the immunological equilibrium of the gut mucosa, generating different gastrointestinal symptoms and reducing the microbiota’s resilience to subsequent disturbances [18,19].

Probiotics are defined by the International Scientific Association for Probiotics and Prebiotics consensus as live microorganisms that, when administered in adequate amounts, provide a demonstrable health benefit to the host [20]. Their effects are predominantly strain-specific and cannot be generalised across different species or even across strains within the same species, though group-level benefits have been documented for certain genera [20,21]. The probiotic field has historically concentrated on bacterial organisms, but growing evidence now supports yeast-based probiotics as a functionally distinct and complementary category.

This review first examines the mechanistic and clinical evidence for bacterial probiotics, subsequently characterises the emerging class of probiotic yeasts, and finally evaluates the rationale and evidence for their complementary use as a strategy to sustain intestinal homeostasis across diverse clinical contexts.

## 2. The Benefits of Probiotic Bacteria

The scientific investigation of bacterial probiotics has a history spanning more than a century [22,23], which started with the observation that consumption of fermented dairy products containing lactic acid-producing bacteria was associated with healthy outcomes in certain populations [23]. This observation led to a research tradition that now includes thousands of clinical trials and has established a range of bacterial genera, mainly *Lacticaseibacillus* and *Bifidobacterium*, as safe and efficacious agents for supporting gut health across diverse clinical contexts [21,24].

Probiotics limit the growth of pathogenic bacteria through several complementary mechanisms. They first compete directly with pathogens for nutrients and for adhesion sites on the intestinal mucosa, reducing the ability of harmful organisms to colonise and persist. In parallel, many probiotic strains produce antimicrobial substances such as bacteriocins, lactic acid, and other organic acids that lower the local pH and create an environment that is hostile to acid-sensitive pathogens but well tolerated by resident commensals. Together, these competitive and biochemical actions establish colonisation resistance, in which a well-structured commensal community physically and chemically excludes invading pathogens and makes intestinal infection much less likely [25,26].

Probiotics contribute actively to maintaining this epithelial barrier by modulating both its cellular and mucus components. They upregulate tight junction proteins, including ZO-1, occludin, and selected claudins, thereby improving junctional organisation and reducing leakage of luminal solutes through the paracellular route. At the same time, they enhance mucin-2 production by goblet cells, which supports renewal and functional quality of the mucus layer rather than merely its thickness, improving its capacity to segregate bacteria and adsorb toxins. Through these coordinated effects on junctional architecture and mucus dynamics, probiotics lessen epithelial exposure to luminal toxins, lipopolysaccharides, and other antigenic stimuli, which in turn stabilises barrier integrity and lowers the risk of systemic translocation of harmful molecules [27].

The third mechanism of probiotics is to modulate the immune system mainly by interacting with intestinal dendritic cells through pattern-recognition pathways such as Toll-like receptor 2 (TLR2), initiating signalling cascades that shape both innate and adaptive responses toward a controlled, protective profile. This interaction favours the differentiation and expansion of regulatory T cells, which secrete interleukin-10 and transforming growth factor-β to maintain immune homeostasis and limit excessive inflammation against commensal antigens. At the same time, probiotics increase the production of secretory IgA at mucosal surfaces, providing targeted immune defence while preserving tolerance, and they downregulate key pro-inflammatory cytokines including interleukin-6, tumour necrosis factor-α, and interleukin-1β, thereby reducing chronic inflammatory activity within the gut [28,29,30].

Finally, probiotics influence host physiology through their metabolite production, both by directly generating bioactive molecules and by stimulating endogenous SCFA-producing bacteria, which amplifies SCFA output beyond what the administered strains could achieve alone. Some probiotic strains also synthesise precursors of neurotransmitters such as serotonin, gamma-aminobutyric acid (GABA), and dopamine, which may cross the intestinal epithelium and access the central nervous system via the systemic circulation. In addition, probiotics produce a range of neuroactive compounds that act on the gut–brain axis through multiple routes, including activation of vagal afferent pathways and modulation of enteric nervous system activity, thereby creating a biochemical bridge between intestinal microbiota composition and neuropsychiatric function [31,32].

Among the many well-characterised bacterial probiotic strains, *Lacticaseibacillus rhamnosus GG* (LGG) stands as one of the most extensively studied, with over three decades of clinical investigation and an evidence base comprising hundreds of randomised controlled trials across gastrointestinal indications including antibiotic-associated diarrhoea (AAD), functional gut disorders, infantile colic, and extraintestinal conditions such as atopic dermatitis [23,33,34,35,36,37,38,39,40,41,42]. Other strains of comparable clinical interest include *Bifidobacterium longum*, *Bifidobacterium infantis*, *Lactobacillus acidophilus*, and *Limosillactobacillus reuteri*, each with strain-specific evidence profiles and distinct niches of activity [21,43,44]. The scientific evidence accumulated for bacterial probiotics reflects decades of investment in their clinical characterisation and established role in supporting gut health. A limitation shared across bacterial strains, however, is their susceptibility to antibacterial agents: under conditions of antibiotic exposure, even well-documented bacterial probiotics may lose viability, limiting their functional availability precisely when gut microbiota support is most needed [45]. This pharmacological constraint, together with their limited capacity to resist environmental stress, has been a principal driver of scientific interest in organisms from an entirely different biological kingdom. Despite their well-established safety profile in healthy individuals, bacterial probiotics are not without risk in specific clinical contexts: in seriously ill, hospitalised, postoperative, and severely immunocompromised patients, administration of viable bacterial strains has been associated with rare but serious adverse events including bacteraemia and sepsis, warranting individualised clinical assessment prior to use in these populations [46].

Beyond the mechanistic evidence reviewed above, the clinical literature on probiotic efficacy has grown substantially over the past two decades, with randomised controlled trials and meta-analyses addressing indications such as AAD, *C. difficile* infection, paediatric acute diarrhoea, irritable bowel syndrome, and inflammatory bowel disease. However, the strength of evidence varies considerably across strains, formulations, and clinical contexts, and current guidance consistently emphasises that probiotic efficacy is strain- and indication-specific rather than a class effect. An important caveat when interpreting this clinical literature is that probiotics are regulated in most jurisdictions as food supplements or dietary supplements rather than as pharmaceutical products and are therefore not subject to the same pre-market requirements for demonstrating efficacy against defined clinical endpoints that apply to medicinal products [47,48]. Consequently, the available trial evidence is characterised by considerable heterogeneity in strain identification, dosing regimens, outcome definitions, and study power, and few trials have been designed with the rigour expected of pivotal registration studies for drugs [49]. This regulatory context does not invalidate the clinical data reviewed above, but it does mean that the strength of evidence supporting any individual probiotic strain for a specific indication remains, on the whole, below the threshold that would be required for a formal therapeutic claim.

## 3. Beyond Bacteria: Probiotic Yeasts

The possibility that eukaryotic fungi could serve as probiotic organisms gained traction with the systematic characterisation of *Saccharomyces boulardii*, a non-pathogenic yeast first isolated from tropical fruit in the early 1920s and subsequently developed as a medicinal preparation [50,51]. The main pharmacological property that distinguished *S. boulardii* from bacterial probiotics was that its cell wall is composed principally of β-glucans, mannoproteins, and chitin [52], none of which are targeted by antibacterial drug classes, conferring intrinsic, structurally determined resistance to antibiotic agents in clinical use [53]. This property was of clinical relevance as it allowed *S. boulardii* to be administered concurrently with antibiotic therapy without loss of viability. Over the following decades, clinical trials established the role of *S. boulardii* in reducing the incidence of AAD, shortening the duration of acute infectious diarrhoea, and preventing recurrence of *Clostridioides difficile*-associated diarrhoea, among others [54].

The clinical and mechanistic success of *S. boulardii* has catalysed wider exploration of the yeast kingdom for probiotic candidates. Among the organisms attracting the most sustained attention is *Kluyveromyces marxianus* [55], a thermotolerant, fast-growing yeast naturally abundant in traditional fermented dairy foods, constituting over 90% of the yeast community in kefir and contributing substantially to artisanal cheese fermentation, with a history of safe human consumption spanning centuries [56]. Like *S. boulardii*, *K. marxianus* has intrinsic resistance to antibacterial agents due to its eukaryotic cell wall, including resistance to β-lactams, fluoroquinolones, aminoglycosides, and sulfonamides [57], and it remains viable throughout the gastrointestinal tract, from the stomach to the colon [58]. Transient colonisation of the colon has been demonstrated both in vitro and in vivo, supporting its capacity to deliver metabolic and immunological signals at the mucosal surface without stable colonisation [57,58,59,60,61,62].

Although *K. marxianus* retains intrinsic fermentative capacity, the observed increases in luminal acetate and propionate concentrations appear to reflect an indirect mechanism predominantly: yeast-mediated remodelling of the colonic microbiota composition that favours the expansion of SCFA-producing taxa, particularly *Bifidobacterium* spp., which are the principal luminal producers of acetate [58]. This pattern parallels the mechanism proposed for *S. boulardii*, which has been shown to augment bacterial butyrate output through interactions with the resident microbiota whose precise mechanistic basis remains incompletely resolved [63]. Its β-galactosidase activity supports the hydrolysis of lactose into absorbable monosaccharides, compensating for the loss of lactase-producing bacteria that frequently accompanies dysbiosis [59,64]. The yeast cell wall releases β-glucan oligomers and mannoprotein fragments that act as soluble immune modulators, engaging C-type lectin receptors on dendritic cells and macrophages. Additionally, *K. marxianus* synthesises B-group vitamins and ergosterol, the principal precursor of vitamin D2 in fungi, contributing to host micronutrient supply during periods of microbial imbalance [65]. It must be emphasised, however, that the evidence supporting these properties in *K. marxianus* derives predominantly from in vitro fermentation systems, colonic model reactors, and animal studies, with only a very limited number of randomised clinical trials. Its alleged contribution to gut homeostasis and its complementary probiotic activity should therefore be regarded as biologically plausible but preliminary hypotheses that require confirmation in adequately powered human studies, in contrast to the more mature clinical evidence available for *S. boulardii* [66].

The safety profiles of both *S. boulardii* and *K. marxianus* are favourable, with adverse events that are typically mild and transient, comprising gastrointestinal adaptation symptoms such as bloating or flatulence [59,67,68,69]. As with all yeast-based probiotics, caution is needed in severely immunocompromised individuals, especially those undergoing intensive chemotherapy or with neutropenia, where the theoretical risk of systemic fungaemia warrants individualised clinical assessment before use [68,69].

Two safety considerations have to be highlighted. First, viable *Saccharomyces* preparations have been causally linked to fungaemia, almost exclusively in critically ill patients with central venous catheters, in intensive-care settings [70], and in the severely immunocompromised, with catheter contamination during sachet handling implicated as a route of translocation; these products are accordingly contraindicated in such populations [71]. Second, bacterial probiotics carry an analogous, if mechanistically distinct, risk of bacteraemia, endocarditis, and sepsis in comparable high-risk groups, including postoperative and short-bowel patients [72]. Contemporary guidance, including the conditional recommendations of the American Gastroenterological Association, therefore advises individualised risk-benefit assessment and caution in hospitalised, catheterised, and immunocompromised patients, while reaffirming the favourable safety profile of both bacterial and yeast probiotics in immunocompetent, ambulatory individuals [73].

## 4. Joining Forces for Gut Health

**Figure 1:**
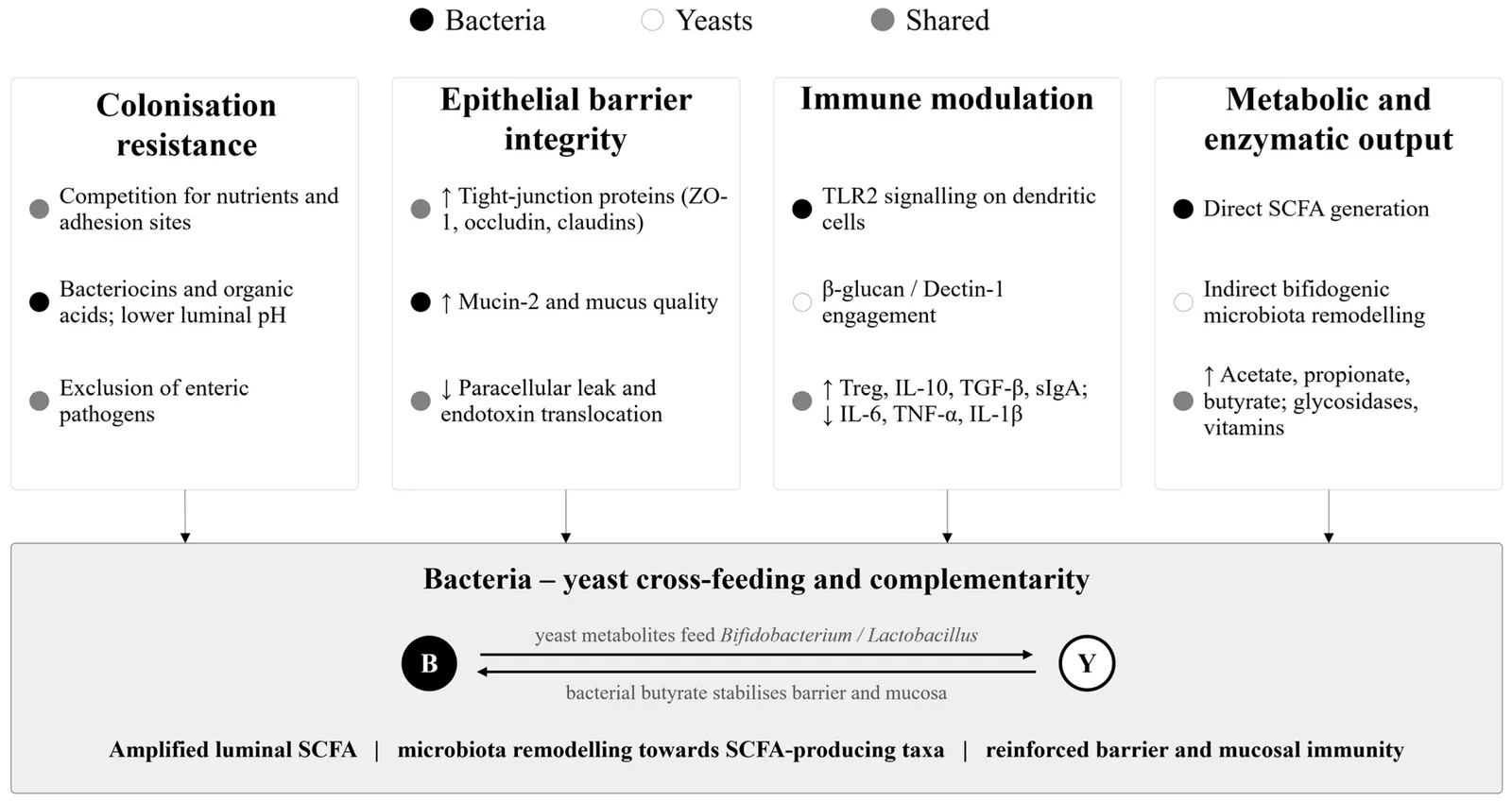
Principal mechanisms of action of probiotic bacteria and yeasts and their complementary interaction. Both organism categories converge on four core axes of intestinal homeostasis (colonisation resistance, epithelial barrier integrity, immune modulation, and metabolic and enzymatic output) through partly distinct molecular routes. Bacterial strains act predominantly through mucosal adhesion, direct SCFA generation, and Toll-like receptor signalling, whereas yeasts contribute antibiotic-resistant activity, β-glucan/Dectin-1 engagement, a broad glycosidase repertoire, and indirect, microbiota-mediated amplification of SCFA output. Bidirectional cross-feeding between the two kingdoms reinforces these effects, yielding amplified luminal SCFA concentrations, microbiota remodelling towards SCFA-producing taxa such as *Bifidobacterium* and *Lactobacillus*, and strengthened barrier and mucosal immune function beyond what either category achieves in isolation. IL, interleukin; sIgA, secretory immunoglobulin A; TGF-β, transforming growth factor-β; TLR, Toll-like receptor; TNF-α, tumour necrosis factor-α; Treg, regulatory T cell; ZO-1, zonula occludens-1.

Bacterial and yeast probiotics occupy distinct ecological niches in the gut, differ in their susceptibility to antibacterial agents, and act through partially divergent mechanisms (Figure 1), but both ultimately serve to stabilise intestinal function and support microbiota homeostasis [74]. These differences support an approach in which the most appropriate probiotic organism is selected according to the prevailing conditions, for example, favouring an antibiotic-resistant yeast during active antibiotic exposure and a bacterial strain with stronger evidence for mucosal recolonisation and barrier repair once treatment is finalised (Table 1). Taking this into account, the concept of “probiotic complementarity” represents a biologically rational framework for organism selection according to the prevailing physiological context, a hypothesis that warrants prospective validation in controlled trials across relevant gastrointestinal indications, rather than substituting for fixed multi-ingredient formulations. Additional supportive measures, such as optimising dietary substrates for beneficial taxa or correcting micronutrient deficits relevant to epithelial repair and immune competence, can then be adapted according to individual needs, with probiotics remaining the central, strain-specific tools for modulating microbiota structure and function [75].

An important fact to consider about bacteria-yeast complementarity is their cross-feeding relationship, which emerges when both categories are present in the intestinal environment. Probiotic yeasts such as *K. marxianus* and *S. boulardii* generate fermentation products and cell wall-derived metabolites, including oligosaccharide fragments and organic acids, that serve as growth substrates for resident *Bifidobacterium* and *Lactobacillus* populations, resulting in enhanced luminal SCFA output that is primarily attributable to the stimulated bacterial communities rather than to direct yeast fermentation [58,76]. In colonic model systems, *K. marxianus* administration has been shown to increase bifidobacterial concentrations in proximal and transverse colon compartments, demonstrating a direct bifidogenic effect that complements the barrier and immune activities of co-administered bacterial strains, although clinical evidence is lacking [58]. Yeast β-galactosidase activity also liberates monosaccharide monomers from lactose that fuel saccharolytic bacteria, while the acidic microenvironment generated by yeast organic acid secretion preferentially supports acid-tolerant commensals such as *Lactobacillus* spp. [77].

Conversely, bacterial butyrate production stabilises tight junction architecture and suppresses conditions that might favour dysbiotic fungal proliferation, showing that the relationship between these two probiotic kingdoms is bidirectional and mutually reinforcing rather than simply additive [6].

Combining probiotics with prebiotic substrates that selectively nourish beneficial microbial populations offers a practical further dimension for optimising a combined bacterial-yeast approach. Fructooligosaccharides (FOS), non-digestible fructan-type dietary fibres found naturally in onions, garlic, artichokes, wheat, and bananas, arrive in the colon largely intact, where they are selectively fermented by bifidobacteria and lactobacilli [78]. Galactooligosaccharides (GOS), enzymatically derived from lactose via transgalactosylation, represent a structurally distinct prebiotic substrate with a comparable fermentation profile; they selectively stimulate the proliferation of *Bifidobacterium* and *Lactobacillus* species in the distal gut, augment luminal SCFA concentrations, and have demonstrated bifidogenic activity in randomised controlled trials across both adult and infant populations [79]. This fermentation expands health-associated microbial taxa, stimulates SCFA production, lowers luminal pH, and limits the growth of opportunistic organisms, amplifying the ecological benefits that bacterial and yeast probiotic strains generate, provided that an adequate fermentable substrate is available to sustain microbial activity [80]. Other specific micronutrients serve additional needs: Zinc, for instance, stabilises tight junctions, mucus, and villus architecture and modulates immune responses [81], while Magnesium participates in over 300 enzymatic reactions relevant to epithelial energy metabolism and barrier assembly [82]. Taken together, these nutritional elements complement the microbiological actions of probiotics by addressing aspects of intestinal homeostasis that fall outside the direct effect of any microbial intervention.

All these facts support the conceptualisation of bacterial and yeast probiotics as distinct tools with overlapping but non-identical capabilities. Bacterial strains offer an extensive clinical evidence base and the most thoroughly characterised mechanisms of mucosal barrier reinforcement and immune regulation. Yeast strains contribute to a biologically distinct mode of probiotic activity that is particularly valuable where antibacterial susceptibility limits the utility of bacterial strains, and they add metabolic and enzymatic functions that extend the scope of probiotic intervention. It is important to distinguish complementarity from synergy in this context [83]. The available data support mechanistic complementarity, that is, non-overlapping and potentially additive functions; genuine clinical synergy, defined as a combined effect exceeding the sum of the individual contributions, has not been demonstrated, because prospective trials directly comparing bacterial monotherapy, yeast monotherapy, and combined administration are lacking [84,85]. The combined approach is therefore best presented as a biologically plausible hypothesis that may complement existing strategies and could provide additional benefit, rather than as an established synergistic therapy.

Because an intact epithelial barrier limits the paracellular passage of luminal antigens and bacterial lipopolysaccharide into the systemic circulation, the barrier-supportive actions described above may carry physiological implications that extend beyond the intestinal lumen [86]. A lower burden of translocated microbial products is mechanistically linked to a reduction in metabolic endotoxaemia, a low-grade inflammatory state associated with less favourable glucose and lipid handling, and thus represents one plausible route connecting intestinal homeostasis to systemic metabolic and cardiovascular physiology [87]. Comparable barrier- and microbiota-mediated principles operate at other mucosal surfaces, including the female genital tract, where the composition and integrity of the local microbial ecosystem contribute to mucosal defence and to the maintenance of physiological homeostasis [88]. These broader connections are presently derived largely from mechanistic and preclinical observations rather than from controlled clinical outcomes, and they should therefore be regarded as a rationale for further investigation rather than as established therapeutic benefits.

The regulatory status of probiotics also has direct implications for their translational use. The term is defined by the International Scientific Association for Probiotics and Prebiotics as live microorganisms that, administered in adequate amounts, confer a health benefit on the host, a definition that presupposes a demonstrated, strain-specific effect [20]. Regulatory frameworks nonetheless diverge across jurisdictions. In the European Union, the European Food Safety Authority has not authorised any health claim for probiotics and regards the word probiotic itself as an implied health claim, so most products are marketed as food supplements without approved claims. In the United States, the Food and Drug Administration regulates such products principally as dietary supplements, whereas live microorganisms intended to prevent or treat disease fall under the more stringent category of live biotherapeutic products and require investigational-new-drug oversight [47]. This distinction between food supplements and medicinal products is decisive for the level of evidence, manufacturing control, and permissible claims applicable to any bacterial-yeast combination such as the one discussed here.

## 5. Conclusions

The available evidence positions bacterial and yeast probiotic organisms as complementary rather than competing strategies for supporting gut microbiota health. Their fundamental differences in cell biology, antibiotic susceptibility, metabolite production, and immune receptor engagement translate into functional profiles that, when combined, address the major axes of intestinal homeostasis, including microbial diversity, SCFA metabolism, epithelial barrier integrity, and mucosal immune calibration, a functional breadth that single-organism interventions may not fully replicate. Bacterial probiotics provide an extensively validated foundation of mucosal protection and immune modulation, with well-characterised strains such as LGG exemplifying the clinical depth achievable through decades of investigation. Yeast probiotics, led historically by *Saccharomyces boulardii* and extended by emerging candidates such as *Kluyveromyces marxianus*, contribute antibiotic-resistant probiotic activity, a broader fermentative and enzymatic repertoire, and cross-feeding effects that actively support commensal bacterial communities, most notably by remodelling colonic microbiota composition in ways that favour the expansion of SCFA-producing taxa, including *Bifidobacterium* and *Lactobacillus species*. This amplifies luminal acetate, propionate, and butyrate concentrations through an indirect, microbiota-mediated mechanism that neither organism category could achieve to the same extent alone. The addition of prebiotic fibres such as FOS, GOS and micronutrients including zinc further enriches this strategy by addressing the substrate availability and structural micronutrient requirements that potentiate probiotic activity.

Prospective, randomised trials directly comparing single-organism probiotic regimens with sequential or concurrent bacterial-yeast protocols across relevant clinical populations are needed to determine whether the mechanistic complementarity described here translates into additive or synergistic clinical benefit, and to define optimal dosing, timing, and formulation strategies. Beyond this central need for head-to-head trials, several knowledge gaps remain: the human evidence for *K. marxianus* is still preliminary; the determinants of trans-kingdom cross-feeding in the human colon are incompletely defined; and optimal strain combinations, dosing, sequencing relative to antibiotic exposure, and long-term safety in vulnerable populations have not been established. Addressing these gaps, rather than assuming a class-wide benefit, should guide the next phase of research into multi-kingdom probiotic strategies.

## Figures and Tables

**Table 1:** Comparative biological properties of probiotic bacteria and yeasts relevant to complementary use.

Feature	Probiotic Bacteria	Probiotic Yeasts
Biological domain	Prokaryote	Eukaryote (fungus)
Cell wall composition	Peptidoglycan, lipoteichoic acids	β-glucans, mannoproteins, chitin
Antibiotic susceptibility	Variable, many strains susceptible to antibacterial agents	Intrinsically resistant to antibacterial drug classes
Primary intestinal niche	Predominantly mucosal; pili-mediated adhesion	Transient; luminal and mucosal compartments
Key enzymatic activities	Bacteriocin production; proteolytic activity	β-galactosidase (*K. marxianus*); broad glycosidase activity (*S. boulardii*, *K. marxianus*); protease secretion (*S. boulardii*)
Principal fermentation products	Lactic acid, acetate	Ethanol (minor); CO_2_; short-chain organic acids (*S. boulardii*: lactic acid, acetic acid); spermine and spermidine polyamines (*S. boulardii*)
Additional bioactive molecules	Bacteriocins; neurotransmitter precursors	B-group vitamins; ergosterol precursors; β-glucan and mannoprotein fragments; yeast-derived metabolites that support enhanced SCFA output (acetate, propionate, butyrate) from co-resident bacteria via microbiota remodelling
Immune receptor engagement	TLR2/TLR4; IgA induction; Treg promotion	Dectin-1/β-glucan recognition; Treg induction (*K. marxianus*); Th1 *(S. boulardii*)
Cross-feeding potential	Lactic acid and SCFA support commensal ecology	Yeast-derived metabolites support *Bifidobacterium* and *Lactobacillus* growth in colonic fermentation models
Representative well-characterised strains	LGG; *B. longum*; *L. acidophilus*	*S. boulardii*; *K. marxianus*

Dectin-1: dendritic cell-associated C-type lectin-1; LGG: *Lacticaseibacillus rhamnosus GG*; Th1: T helper type 1; TLR: Toll-like receptor; SCFA: short-chain fatty acid; Treg: regulatory T cell.

## Data Availability

No new data were created or analyzed in this study. Data sharing is not applicable to this article.

## Abbreviations

The following abbreviations are used in this manuscript:

AAD	Antibiotic-associated diarrhoea
CO_2_	Carbon dioxide
FOS	Fructooligosaccharides
GABA	Gamma-aminobutyric acid
GOS	Galactooligosaccharides
GPR41	G-protein-coupled receptor 41
GPR43	G-protein-coupled receptor 43
GPR109a	G-protein-coupled receptor 109a
IgA	Immunoglobulin A
IL	Interleukin
IL-1β	Interleukin-1β
IL-6	Interleukin-6
IL-10	Interleukin-10
LGG	*Lacticaseibacillus rhamnosus* GG
SCFAs	Short-chain fatty acids
sIgA	Secretory immunoglobulin A
TGF-β	Transforming growth factor-β
Th1	T helper type 1
TLR	Toll-like receptor
TLR2	Toll-like receptor 2
TLR4	Toll-like receptor 4
TNF-α	Tumour necrosis factor-α
Treg	Regulatory T cell
ZO-1	Zonula occludens-1
